# Extracellular renalase protects cells and organs by outside‐in signalling

**DOI:** 10.1111/jcmm.13062

**Published:** 2017-02-26

**Authors:** Yang Wang, Robert Safirstein, Heino Velazquez, Xiao‐Jia Guo, Lindsay Hollander, John Chang, Tian‐Min Chen, Jian‐Jun Mu, Gary V. Desir

**Affiliations:** ^1^ Department of MedicineVeterans Affairs Connecticut Healthcare SystemYale UniversityNew HavenCTUSA; ^2^ Department of CardiologyFirst Affiliated Hospital of Medical SchoolXi'an Jiaotong UniversityXi'anChina; ^3^ Department of SurgeryUniversity of ConnecticutFarmingtonCTUSA

**Keywords:** renalase, survival factor, cell signalling, ischaemic injury, immune‐oncology, macrophages

## Abstract

Renalase was discovered as a protein synthesized by the kidney and secreted in blood where it circulates at a concentration of approximately 3–5 μg/ml. Initial reports suggested that it functioned as an NAD(P)H oxidase and could oxidize catecholamines. Administration of renalase lowers blood pressure and heart rate and also protects cells and organs against ischaemic and toxic injury. Although renalase's protective effect was initially ascribed to its oxidase properties, a paradigm shift in our understanding of the cellular actions of renalase is underway. We now understand that, independent of its enzymatic properties, renalase functions as a cytokine that provides protection to cells, tissues and organs by interacting with its receptor to activate protein kinase B, JAK/STAT, and the mitogen‐activated protein kinase pathways. In addition, recent studies suggest that dysregulated renalase signalling may promote survival of several tumour cells due to its capacity to augment expression of growth‐related genes. In this review, we focus on the cytoprotective actions of renalase and its capacity to sustain cancer cell growth and also the translational opportunities these findings represent for the development of novel therapeutic strategies for organ injury and cancer.

## Introduction

Renalase, first discovered in 2005, is a flavin/adenine/dinucleotide‐dependent amine oxidase that is secreted into the blood by the kidneys and is suggested to participate in catecholamine metabolism 1. It is also expressed in heart, intestine, liver, skeletal muscle and endothelium 2, 3. Certain single nucleotide polymorphisms present in the renalase gene have been associated with an increased risk of developing essential hypertension, chronic kidney disease (CKD), heart disease, diabetes and stroke 4, 5, 6, 7. Previous evidence showed that recombinant renalase exerts powerful and rapid hypotensive effects in rodents; this effect was suggested to be mediated by the degradation of circulating catecholamines, which could decrease cardiac contractility and heart rate 8, 9.

Renalase is secreted in blood, and its levels are regulated by at least three factors: renal function, renal perfusion and catecholamine levels. Plasma renalase levels measured by Western blot suggest a direct relationship with glomerular filtration rate and renal mass, resulting in renalase deficiency as renal function declines 1, 10. Subtotal nephrectomy (5/6 Nx) in rats results in decreased plasma renalase, thus suggesting that renal function and renal mass are important determinants of steady‐state renalase levels 10. In a model of unilateral renal artery stenosis, renalase expression and secretion are decreased in the ischaemic kidney, thus implicating changes in renal perfusion as a determinant of renalase secretion 11. In the isolated perfused rat kidney model, catecholamine infusion stimulates renalase secretion into the renal vein as well as its activation 11, thus linking renalase secretion to prevalent catecholamine levels. Taken together, these data suggest that the kidney regulates renalase secretion in plasma, both in basal and in stimulated states.

Remarkable progress has been achieved over the past 3 years in understanding the cellular actions of renalase, its pathophysiology and potential therapeutic utility. The protein exerts its cytoprotective effects by interacting with a plasma membrane receptor PMCA4b (and possibly another receptor) independent of its catalytic activity 12, 13. This review will summarize new findings indicating that renalase functions as a cytokine that protects cells and organs exposed from ischaemic and toxic injury. A second focus will be to explore renalase's role as a survival factor for tumour cells because new evidence suggests that dysregulated renalase signalling can promote the survival and growth of cancer cells in an autocrine or paracrine manner.

## The mechanism of renalase's cytoprotective properties

Initially, a number of studies showed that renalase expression had a major effect on catecholamine metabolism *in vivo*. Its expression and activity were down‐regulated by increased dietary phosphate 14. A renalase‐deficient mouse (renalase KO) model was used to explore the mechanisms mediating renalase's effect on phosphate (PO4‐) excretion and revealed that renalase deficiency is associated with increased renal dopamine (DA) synthesis, stimulated PO4‐excretion and moderately severe hypophosphatemia. Of note, the signal to increase renal DA synthesis is strong as it overcomes a compensatory increase in catechol‐O‐methyltransferase (COMT) activity 15. Plasma catecholamine levels are modulated by renalase expression 16. Compared to wild‐type (WT) mice, KO mice had increased plasma levels of epinephrine (EPI), norepinephrine (NE) and DA and increase in urinary DA/L‐3,4‐dihydroxyphenylalanine (L‐DOPA) ratios without changes in renal tubular aromatic‐L‐amino acid decarboxylase activity. The *in vivo* administration of recombinant renalase to KO mice led to a significant decrease in plasma levels of EPI, DA and L‐DOPA as well as in urinary excretion of EPI, DA and DA/L‐DOPA ratios 16.

Renalase was first shown to metabolize NADH by Farzaneh‐Far *et al*. 17, and it was suggested that H_2_O_2_ and superoxide anion generated as by‐products of NADH oxidation could oxidize EPI to adrenochrome. Aliverti and Moran *et al*. 18, 19, 20, 21 confirmed that EPI was bound to renalase, but could not demonstrate EPI oxidation. Moran then proposed that renalase functioned as an oxidase/anomerase, using molecular oxygen to convert β‐NAD(P)H into β‐NAD(P)+, with hydrogen peroxide as a reaction by‐product 18, 19, 20, 21. This assertion turned out to be incorrect, and they subsequently provided evidence suggesting that renalase converts endogenous dihydro forms of β‐NAD(P)H, which are postulated to inhibit intracellular enzymes, to metabolically available, non‐inhibitory β‐NAD(P)H 21, 22.

Fedchenko *et al*. 23 purified renalase from urine of healthy volunteers and could not detect the N‐terminal signal peptide and FAD. Computer‐aided analysis further showed that the removal of this peptide results in inability of the truncated renalase to bind the FAD cofactor. They also found that human recombinant renalase‐1 secreted by HEК293T cells also lacks the N‐terminal signal peptide. These results suggest renalase's signal peptide is cleaved upon secretion and that extracellular (blood and urine) renalase acts in a FAD‐independent manner 24. However, the possibility of additional proteolytic processing of extracellular renalase should be considered 25.

## Extracellular renalase protects against renal injury

**Figure 1:**
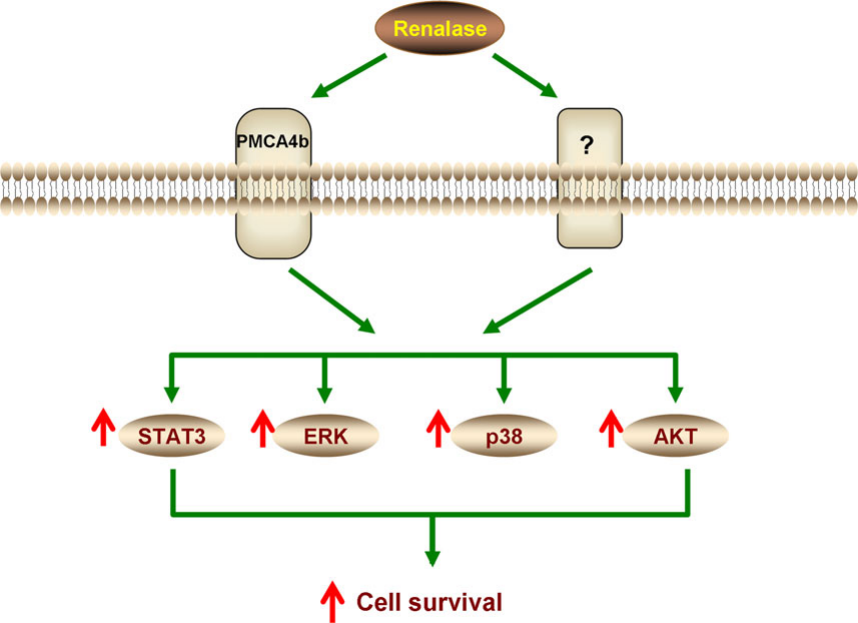
Working model of renalase as a cytokine. Extracellular renalase interacts with a plasma membrane receptor to activate signal transducer and activator of transcription (STAT3) and mitogen‐activated protein kinase (MAPK) pathways and increase cell survival. PMCA4b: plasma membrane calcium ATPase isoform 4b, AKT: protein kinase B, ERK: extracellular signal‐regulated kinase, p38: p38 mitogen‐activated kinase.

Renalase‐deficient mice subjected to renal ischaemia reperfusion (I/R) developed significantly worse renal tubular necrosis, inflammation and apoptosis, and administration of recombinant renalase ameliorated these changes in ischaemic acute kidney injury (AKI) 26. Renalase also protected against cisplatin‐mediated AKI. Compared to wild type, renalase‐deficient mice exposed to cisplatin developed more severe renal injury as assessed by serum creatinine, renal injury score, of apoptosis and macrophage infiltration. Administration of recombinant human renalase protected human proximal tubular (HK‐2) cells against cisplatin‐ and hydrogen peroxide‐induced toxic injury and prevented ischaemic injury in mice 13. A key finding in this study is that renalase activates a receptor‐mediated, prosurvival signalling cascade, with activation of protein kinase B (AKT), extracellular signal‐regulated kinase (ERK), p38 mitogen‐activated kinase (p38), B cell lymphoma 2 and inhibition of c‐Jun N‐terminal kinase 13. A 20‐amino acid peptide (RP‐220), conserved in all known renalase isoforms, but lacking detectable oxidase activity, rapidly signalled *via* MAPKs and fully mimicked the protective effect of renalase in cells exposed to cisplatin 13. This was used in cross‐linking studies to identify the plasma membrane calcium ATPase PMCA4b as the renalase receptor that mediates renalase‐dependent cell signalling and cytoprotection 12. PMCA4b is a low‐capacity calcium pump that regulates local rather than bulk calcium concentration and functions primarily as part of a signalling complex 27. These results indicate that cytoprotective actions of renalase against renal ischaemic and toxic injury are mediated by outside‐in signalling through PMCA4b to activate the PI3K/AKT and MAPK pathways (Fig. 1).

An *in vivo* by a rat model of renal I/R injury after remote preconditioning (IPC) also highlights renalase role. Remote preconditioning (RPC) refers to the process by which short periods of induced ischaemia of an organ or limb confers protective effects to other tissues 28. IPC before renal I/R injury significantly reduced renal tubular inflammation, necrosis and oxidative stress partly by up‐regulating renalase expression 29. Inhibition of renalase with its monoclonal antibody attenuated such effects, suggesting that the renoprotective effect of renal IPC is partly mediated by renalase 29. In addition, Zhao and colleagues reported that pre‐treatment with renalase attenuated the deterioration of renal function, tubular necrosis, oxidative stress, apoptosis and inflammation in rats with contrast‐induced nephropathy (CIN) 30. Renalase also protected HK‐2 cells against the cytotoxicity of ioversol and suppressed caspase‐3 activity, oxidative stress and apoptosis induced by H_2_O_2_, suggesting that renalase protected CIN in rats through antioxidation, anti‐apoptosis and anti‐inflammation mechanisms 29. More recently, the same authors reported that RPC‐induced renoprotection in CIN is dependent on increased renalase expression *via* activation of the TNF‐α/NF‐κB pathway 31. They found that RPC prevented renal function decline, attenuated tubular injury and reduced oxidative stress and inflammatory response in the kidney. These beneficial effects were abolished by silencing of renalase with siRNA 31. This study establishes a vital role of renalase in RPC in the kidney and establishes a previously unknown mechanism whereby induced peripheral ischaemia leads to the release of TNF‐α which then induces RPC in the kidney by a NF‐κB‐mediated up‐regulation of renalase expression 32.

## Extracellular renalase protects against cardiac injury

In addition to its effects on kidney injury, renalase protects against acute cardiac ischaemia and prevents the development of cardiac hypertrophy. The degree of myocardial necrosis caused by acute ischaemia was threefold more severe in the global renalase KO, compared to WT, and the phenotype could be rescued by the administration of recombinant renalase 8. Du *et al*. 33 use cardiac injection renalase siRNA in C57BL/6 to specifically down‐regulate expression in the heart and found that decreased cardiac renalase was associated with more severe I/R injury as demonstrated by increased infarct size and decreased ejection fraction (EF). The administration of recombinant renalase reduced the infarct area and prevented a fall in EF. They further identified renalase was a novel target gene of hypoxia‐inducible factor‐1 alpha (HIF‐1α) 33. The protective and prosurvival role of renalase was also established by the study of Li *et al*. 34, in which they demonstrated that renalase protected myocardial cells from I/R injury through an anti‐apoptotic and anti‐inflammatory mechanism.

Renalase protects against the development of cardiac hypertrophy associated with CKD. Baraka *et al*. 35 administered recombinant renalase to rats undergoing 5/6 nephrectomy (Nx) and observed a significant decrease in left ventricular (LV) hypertrophy, LV hydroxyproline concentration and LV papillary muscle dysfunction. Yin *et al*. 36 utilized a similar animal model and reported that renalase reduced proteinuria, glomerular hypertrophy and interstitial fibrosis, as well as significantly decreased expression of genes for fibrosis markers, proinflammatory cytokines and nicotinamide adenine dinucleotide phosphate (NADPH) oxidase components. Administration of recombinant renalase attenuated the development of hypertension, cardiomyocytes hypertrophy and cardiac interstitial fibrosis and prevented cardiac remodelling through inhibition of profibrotic genes expression and phosphorylation of ERK‐1/2 36. Together, these findings strongly suggest that renalase protects against acute cardiac injury and mitigates the development of cardiac hypertrophy associated with CKD.

## Extracellular renalase protects against acute pancreatitis

Similar to its protective effect in kidney and heart, renalase ameliorates the course of acute pancreatitis. Genetic deletion of renalase is associated with more severe acute pancreatitis, and the administration of exogenous renalase (either prophylactically or therapeutically) dramatically reduced the severity of acute pancreatitis 37. Plasma renalase decreases significantly at the onset of acute pancreatitis suggesting it could serve as a diagnostic or predictive marker. Mechanistic studies revealed that renalase may affect Ca^2+^ signalling by binding to and activating PMCA4b, a key mechanism for Ca^2+^ efflux from the acinar cell 37. These data suggest that renalase protects against acute pancreatitis by modulating calcium transport and that the administration of recombinant renalase may prove to be a valuable therapeutic option for patients with acute pancreatitis.

There is strong evidence that renalase exerts a powerful cytoprotective function in acute tissue and organ injury states, including AKI, cardiac injury and acute pancreatitis. Whether renalase‐dependent cytoprotection is mediated exclusively by outside‐in signalling driven by extracellular renalase remains to be proven. And additional studies should be carried out to establish the relative contribution of extra‐ and intracellular renalase to organ protection against acute injury. Furthermore, although the mechanism of action of intracellular renalase has been assumed to rely on its putative enzymatic function, preliminary results suggest that intracellular renalase also displays transcription factor‐like properties such as nuclear translocation and a positive feedback loop with STAT3, and further studies on the function of intracellular renalase are warranted.

## Dysregulated extracellular renalase signalling promotes tumour growth

As renalase functions as a survival factor by activating ERK, AKT and signal transducer and activator of transcription (STAT3) and appears to participate in a positive feedback loop with STAT3 13, 38, we explored whether renalase expression and signalling provided a survival advantage to cancer cells.

We first examined renalase expression in commercially available cDNA arrays at various tumours and found that renalase expression was significantly increased in cancers of the pancreas, bladder and breast and in melanoma. We focused on pancreatic cancer due to its particularly poor survival and limited therapeutic options. In a cohort of patients with pancreatic ductal adenocarcinoma (PDAC), we found that renalase levels significantly increased (>twofold) in PDAC tumours compared to the adjacent non‐tumour pancreatic tissue, and overall survival was inversely correlated with renalase expression in the tumour mass 39. *In vitro* studies indicate that recombinant renalase increased PDAC cells survival rate by twofold to fivefold. Inhibition of renalase signalling using siRNA or inhibitory antirenalase antibodies decreased the viability of cultured PDAC cells. Renalase also improved PDAC survival in an ERK‐ and STAT3‐dependent manner because pre‐treating with U0126, an inhibitor of the MAP kinase MEK1 and AG490, a compound inhibits JAK2, Erk2 and STAT3 activity, abrogated its protective effect. In two xenograft mouse models, either the renalase monoclonal antibody m28‐Renalase or shRNA knockdown of renalase inhibited PDAC growth. Inhibition of renalase caused tumour cell apoptosis and cell cycle arrest 39.

**Figure 2:**
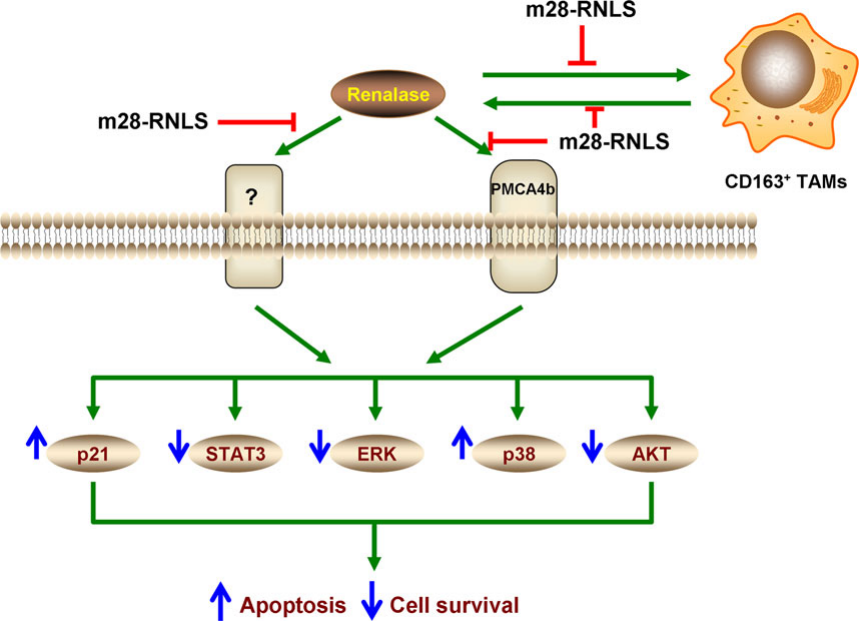
Proposed mechanism of action of m28‐RNLS. Inhibition of renalase signalling decreases renalase secretion from CD163+ TAMs and inhibits RNLS signalling in melanoma cells, all leading to a significant increase in p21 and reduction in MAPK pathways, and cellular apoptosis. m28‐RNLS: antirenalase monoclonal antibody, PMCA4b: plasma membrane calcium ATPase isoform 4b, STAT3: signal transducer and activator of transcription, AKT: protein kinase B, ERK: extracellular signal‐regulated kinase, p38: p38 mitogen‐activated kinase.

We next examined the role of renalase in promoting the growth of melanoma, a disorder in which the MAPK, PI3K and JAK/STAT pathways are regulated abnormally, and found that renalase expression was increased progressively from normal skin to benign nevi to primary malignant melanoma and finally to metastatic melanoma. Significant renalase expression was detected in CD163+ (M2‐like) tumour‐associated macrophages (TAMs). In clinical specimens, renalase expression in the tumour correlated inversely with disease‐specific survival, suggesting a pathogenic role for renalase. Treatment with recombinant renalase in melanoma cells showed increased cell counts and increased percentage of live cells. Attenuation of renalase expression and signalling by RNAi, or the inhibitory renalase monoclonal antibody m28‐RNLS, decreased cell proliferation and increased apoptosis in tumour cells. m28‐RNLS blocked tumour growth in an immune competent murine xenograft model 40. Mechanistic investigations showed that PMCA4b mediated renalase‐dependent STAT3 and ERK1/2 phosphorylation in melanoma cells. Dysregulated renalase signalling appears to promote macrophage polarization towards a tumour promoting, M2‐like phenotype (Fig. 2) 40. Based on these findings, we propose that abnormal up‐regulation of renalase signalling favours cancer cell survival and promotes tumour growth. We further propose that drugs designed to inhibit renalase signalling may provide novel therapeutic options in cancer.

## Future perspectives

It will be important to develop standardized validated methods to assess renalase activity and levels in various fluids, including urine, and to resolve the discrepancies in blood levels based on Western blots and ELISA in patients with kidney disease. We have discussed this issue in detail in a prior review article 5. Another priority should be to define the relative contribution of intracellular and extracellular renalase to the protein's overall cytoprotective properties. As intracellular renalase contained a clearly detectable N‐terminal peptide and FAD cofactor, which appears to be absent in extracellular renalase, the molecular mechanisms of intracellular renalase will need to be examined further. Specifically, does it act as an enzyme, a signalling molecule or a transcription factor?

Another key goal will be to define the therapeutic utility of renalase‐based therapy for acute tissue and organ injury. The data in acute kidney and cardiac injury are compelling, as they are for acute pancreatitis. Conversely, the inhibition of renalase signalling may provide new therapeutic opportunities for cancer patients, and the developments of humanized and fully human antirenalase antibodies are currently underway. It will be interesting to test whether inhibition of extracellular renalase signalling by renalase antibodies affects intracellular renalase levels. We will also need to assess the potential side‐effects of antirenalase therapy and develop protocols that provide favourable risk benefit ratios.

## Conclusions

Our understanding of the physiology and pathophysiology of renalase has expanded dramatically over the past 3 years. The discovery that, independent of its intrinsic enzymatic properties, renalase functions as a cytokine with general cytoprotective actions, by interacting with plasma membrane receptor PMCA4b to activate cell survival pathways, is a major step forward. These findings could serve as the basis for further work aimed at elucidating key molecular mechanisms in injury states such as AKI, myocardial infarction and acute pancreatitis and for exploring the therapeutic utility of acute and short‐term administration of renalase to treat these disorders. Furthermore, dysregulated renalase signalling provides a survival advantage to cancer cells, and inhibition of renalase signalling using inhibitory antibodies not only has direct cytotoxic effect on tumour cells, but also modulates the immune response to tumour by shifting TAMs from a M2‐like tumour promoting phenotype to a M1‐like tumour inhibitory one. These findings suggest that renalase inhibition may provide novel therapeutic options for patients with pancreatic cancer and melanoma and perhaps other tumours.

## Conflicts of interest

G Desir is a named inventor on several issued patents related to the discovery and therapeutic use of renalase. Renalase is licensed to Bessor Pharma, and G Desir holds an equity position in Bessor and its subsidiary Personal Therapeutics.
